# MicroRNA Profiling of Activated and Tolerogenic Human Dendritic Cells

**DOI:** 10.1155/2014/259689

**Published:** 2014-04-01

**Authors:** Zuzana Stumpfova, Renata Hezova, Albano Carlo Meli, Ondrej Slaby, Jaroslav Michalek

**Affiliations:** ^1^Department of Pharmacology, Faculty of Medicine, Masaryk University, Kamenice 5, 62500 Brno, Czech Republic; ^2^CEITEC, Masaryk University, Kamenice 5, 62500 Brno, Czech Republic; ^3^Masaryk Memorial Cancer Institute, Zlutý Kopec 7, 65653 Brno, Czech Republic; ^4^Department of Biology, Faculty of Medicine, Masaryk University, Kamenice 5, 62500 Brno, Czech Republic; ^5^INSERM U1046, University of Montpellier I and II, 371 avenue du Doyen G. Giraud, 34295 Montpellier, France

## Abstract

Dendritic cells (DCs) belong to the immune system and are particularly studied for their potential to direct either an activated or tolerogenic immune response. The roles of microRNAs (miRNAs) in posttranscriptional gene expression regulation are being increasingly investigated. This study's aim is to evaluate the miRNAs' expression changes in prepared human immature (iDCs), activated (aDCs), and tolerogenic dendritic cells (tDCs). The dendritic cells were prepared using GM-CSF and IL-4 (iDC) and subsequently maturated by adding LPS and IFN-**γ** (aDC) or IL-10 and TGF-**β** (tDC). Surface markers, cytokine profiles, and miRNA profiles were evaluated in iDC, tDC, and aDC at 6 h and 24 h of maturation. We identified 4 miRNAs (miR-7, miR-9, miR-155 and miR-182), which were consistently overexpressed in aDC after 6 h and 24 h of maturation and 3 miRNAs (miR-17, miR-133b, and miR-203) and miR-23b cluster solely expressed in tDC. We found 5 miRNAs (miR-10a, miR-203, miR-210, miR-30a, and miR-449b) upregulated and 3 miRNAs downregulated (miR-134, miR-145, and miR-149) in both tDC and aDC. These results indicate that miRNAs are specifically modulated in human DC types. This work may contribute to identifying specific modulating miRNAs for aDC and tDC, which could in the future serve as therapeutic targets in the treatment of cancer and autoimmune diseases.

## 1. Introduction

Different cell types constitute a group termed as dendritic cells (DCs) that modulate the balance between innate and adaptive immunity. Basically, DCs adjust T lymphocytes either to activate or suppress a specific immune response in the body. Novel medical therapy strategies aim to exploit DCs abilities to restore body homeostasis. The particular DCs subsets are shaped by various maturation stimuli that affect DCs during their life cycle. Recently,* in vitro* prepared DCs have been cultured with different immunomodulatory agents, including immunoactivators associated with microbial patterns (e.g., bacterial peptidoglycan or lipopolysaccharide (LPS)) and proinflammatory mediators (e.g., IL-6, IFN-*γ*) [1, 2]. In contrast, tolerogenic DCs (tDCs) induction protocols include a combination of IL-10 and/or TGF-*β* or agents such as 1*α*,25-dihydroxyvitamin D3 or dexamethasone [3, 4]. As a result, fully matured activated DCs (aDCs) produce high levels of proinflammatory cytokines such as IL-6, IL-12, and IFN-*γ*, upregulate coreceptors CD80/CD86, and have become a promising candidate for modern anticancer therapies.

On the other hand, tDCs perpetuate a steady state characterized by antigen presentation without T cell activation. In cell-to-cell interactions, tDCs convert naïve T cells to regulatory T lymphocytes, induce anergy in autoreactive T cells, and expand naturally occurring T regulatory and T suppressor lymphocytes [5, 6]. For these reasons, tDCs may be used in autoimmune disorders or graft rejection therapies. Since DCs were first identified [7, 8], significant progress [9–11] has been achieved in understanding their biology.

In our study we focused on the first 6 h of maturation as it has been shown that DCs cease secretion of crucial immunostimulatory factors after 24 h of cultivation [12, 13]. External stimuli are transferred to the DCs intracellular space and the end point of intracellular process results in gene expression that is posttranscriptionally regulated by microRNAs (miRNAs).

The miRNAs are small noncoding RNAs that inhibit their target mRNAs translation by binding to them [14]. They are essential in a variety of developmental and physiological processes [14] and play a crucial role in cancerogenesis, homeostasis maintenance, immune cell development and differentiation, antibody production, and inflammatory mediator release [15–18]. Each miRNA may control the expression of hundreds of target genes and also several miRNAs directly regulate one mRNA. Therefore, different miRNAs may influence target genes coordinately or synergistically [19, 20] and regulate fine-tune immune response, including DCs function [15–18, 21].

In this study, we focused on the first 6 h of maturation of DCs and compared DCs phenotypes using cytokine production and cell surface markers as well as miRNA profiles. As in previous reports [1, 2, 22], our results indicate that LPS and IFN-*γ* induce DCs activation whereas TGF-*β* and IL-10 lead to DCs tolerogenic character during maturation* in vitro*. Upon maturation, we identified 4 miRNAs (miR-7, miR-9, miR-155, and miR-182) consistently upregulated in aDCs and 4 other miRNAs (miR-17, miR-133b, miR-203, and miR-23b) in tDCs. We also found 4 miRNAs (miR-10a, miR-203, miR-210, and miR-449b) upregulated and 3 miRNAs downregulated (miR-134, miR-145, and miR-149) in both tDCs and aDCs. To the authors' best knowledge, this is the first time the miRNA profile of human tDCs generated with IL-10 and TGF-*β* has been described.

In conclusion, this work may contribute to identifying key tolerogenic miRNAs as potential therapeutic targets in the treatment of autoimmune disease or as immunomodulators after organ transplantation and for activating miRNAs to characterize and/or activate DCs used for cancer immunotherapy.

## 2. Materials and Methods

### 2.1. Preparation of Human Dendritic Cells

Buffy coats from healthy donors were obtained from the Department of Transfusion Medicine and Blood Bank, University Hospital (Brno, Czech Republic). All subjects' blood samples were taken after signing an informed consent form approved by the local ethical committee. Peripheral blood mononuclear cells (PBMCs) were isolated using density gradient centrifugation on Histopaque (Nycomed Pharma, Oslo, Norway). The PBMCs (1 × 10^6^ cells/mL) were placed in a 5 mL Petri dish (Nunclon) for 2 h plastic adherence. To obtain iDCs, adherent cells were cultured in 5 mL of complete media X-VIVO 10 (BioWhittaker, Walkersville, USA) supplemented with 2 mM glutamine (Bio Whittaker), 3% heat inactivated human AB serum (SigmaAldrich, USA), 800 UI/mL GM-CSF (PeproTech, USA), and 500 UI/mL IL-4 (Prospec, Israel) for 6 days, media was changed after 3 days. On day 6, iDCs were matured for a further 6 h or 24 h by 50 ng/mL IFN-*γ* (ProSpec, Israel) and 200 ng/mL LPS (Calbiochem, MA, USA). The resulting DCs were called aDCs. tDCs were matured using 1 ng/mL IL-10 (PeproTech, USA) and 2 ng/mL TGF-*β* (PeproTech, USA) for the same time period as the aDCs. The DCs measurements and harvests were carried out on day 6 of cultivation (0 h) and at 6 h and 24 h of maturation.

### 2.2. Flow Cytometric Analysis of DCs Surface Markers

Before maturation (0 h) and after 6 h and 24 h of maturation, the harvested DCs were washed. Staining was performed with fluorescent antibody: anti-HLA-DR-PC7, anti-CD80-FITC, anti-CD86-PE (Beckman Coulter Inc., Immunotech, France), and anti-CD83-APC (BD Pharmingen, BD Bioscience, NJ, USA). The DCs were measured using a FACSCanto flow cytometer (Becton Dickinson, NJ, USA). Data were analyzed using BD FACSDiva 6.0 software.

### 2.3. Cytokines Profiles in DCs Culture Media

The DCs culture media supernatants collected after 6 days of cultivation (0 h) and 6 h and 24 h of maturation were measured to obtain the levels of the following cytokines: IL-6, IL-10, IL-12p70, IFN-*γ*, and TNF-*α*. Human simplex kits and human basic kit, such as the bead based analyte detection assays, were used for the quantitative detection of the previously mentioned cytokines (Bender MedSystems GmbH, Austria). The samples were assessed according to the manufacturer's protocol. Analyte concentrations were proportional to the fluorescent intensity measured on a flow cytometer system FACSArray (BD Biosciences, NJ, USA). Data were acquired using BD FACSArray System Software version 1.0.4 (BD Biosciences, NJ, USA) and analyzed using FlowCytomix Pro 2.4.

### 2.4. RNA Isolation and TaqMan Low Density Array (TLDA)

Total RNA enriched with small RNAs was isolated using the mirVANA miRNA Isolation Kit (Ambion Inc., Austin, TX, USA) according to the manufacturer's protocol. Total RNA concentration and purity were controlled using UV spectrophotometry (A260/A280 < 2.0) using a NanoDrop ND-1000 (Thermo Scientific, Wilmington, DE, USA). 100 ng of total RNA was reverse-transcribed into cDNA using a Multiplex RT set pool (Applied Biosystems, CA, USA) and loaded onto a TLDA Human miRNA Panel containing 384 wells (368 TaqMan MicroRNA Assays enabling the simultaneous quantification of 365 human miRNAs and 3 endogenous controls) according to the manufacturer's protocol. Quantitative miRNA expression data were acquired and normalized using the ABI Prism 7900HT Sequence Detection System (Applied Biosystems, CA, USA).

### 2.5. Expression Data Analysis

Data are presented as the mean values ± SD of individual experiments. Because of the nonparametric distribution of data, the Mann-Whitney *U* test was performed to assess statistical differences between the two experimental groups. In all cases a *P* value < 0.05 was considered significant. The miRNA gene expression values were normalized according to endogenous control RNU6B and relative expression values were obtained using the ΔCt method with SDS software v 2.3 (Applied Biosystems, CA, USA). The relative target miRNA expression levels were determined by the equation 2^−ΔCt^, in which ΔCt was calculated as follows: ΔCt = CtmiR-of-interest − Ct RNU6B. Different miRNA expression between the groups was identified according to fold change. Fold change cut-off was 2-fold, if the changes were observed in both cell samples simultaneously. The miRNA expression levels were visualized through heat maps (TIGR MultiExperiment Viewer 4.0, The Institute for Genomic Research, USA), dendrograms, and column charts. The Spearman correlation test was used to correlate miRNA expression data with interleukin production.

## 3. Results

### 3.1. Maturation with IL-10 and TGF-*β* Leads to Decreased CD80 and CD83 Expression

**Figure 1:**
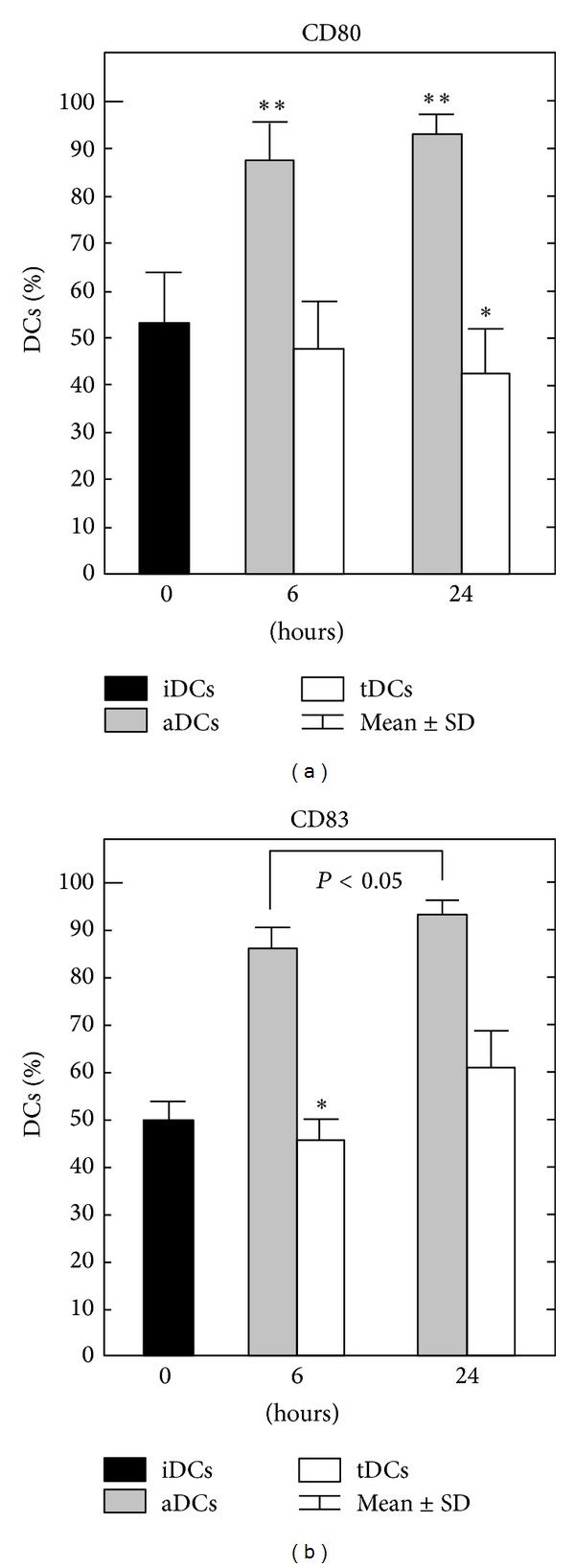
Expression of surface markers CD80, CD83 on DCs after maturation. Immature iDCs were cultured with GM-CSF (800 UI/mL) and IL-4 (500 UI/mL) for 6 days. On day 6 iDCs media were replaced with media containing LPS and IFN-*γ* (aDCs) or with media containing IL-10 and TGF-*β* (tDCs) for the next 6 h or 24 h of maturation. DCs were subsequently washed and the surface markers CD80, CD83, CD86, and HLA-DR were measured using flow cytometer. The results are expressed as mean ± SD of eight individual experiments. *P < 0.05; **P < 0.001 (significant difference to iDCs). Exact *P* values are given in Section 3.

To measure the effect of maturation cocktails on DCs, we analyzed the profiles of T cell stimulator molecules HLA-DR and induced costimulatory (CD80/CD86) and maturation (CD83) markers using FACS analysis. The aDCs treated with LPS and IFN-*γ* showed a classical mature DCs phenotype that was already evident at 6 h of maturation. The aDCs expressed a significantly high DCs differentiation marker CD83 (*P* < 0.001) and costimulatory molecule CD80 (*P* < 0.001) levels compared with untreated iDCs. Both markers were even more expressed at 24 h aDCs (Figure 1). The IL-10 and TGF-*β* caused significant CD80 downregulation at 24 h of maturation in tDCs compared to iDCs (*P* < 0.05). We found a significant decrease of CD83 in tDCs compared to iDCs only at 6 h (*P* < 0.05, Figure 1). CD83 in tDCs was retained downregulated in comparison to both 6 and 24 h aDCs (*P* = 0.007, Figure 1). However, we found gradual reduction of CD80 expression from 6 to 24 h tDCs compared to 6 h (*P* < 0.0001) and 24 h (*P* < 0.000001) aDCs (Figure 1). CD86 costimulatory receptor and HLA-DR molecule did not show significant differences between tDCs and aDCs (*data not shown*).

### 3.2. Cytokine Profiles

**Figure 2:**
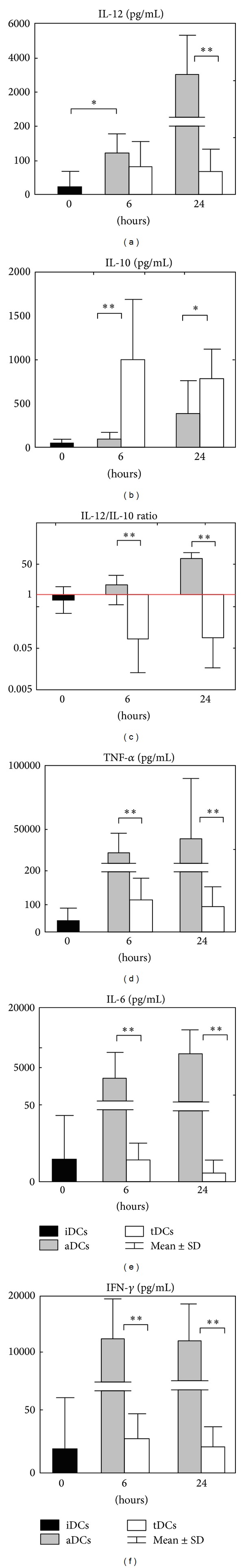
Quantification of cytokines in DCs upon maturation. Immature iDCs were cultured with GM-CSF (800 UI/mL) and IL-4 (500 UI/mL) for 6 days. On day 6, iDCs media were replaced with media containing LPS and IFN-*γ* (activated DCs: aDCs) or with media containing IL-10 and TGF-*β* (tolerogenic DCs: tDCs) for next 6 h or 24 h maturation. The cytokines concentrations were measured in collected DCs culture media by bead based FACSArray. (a) Concentration of IL-12p70 (pg/mL). (b) Concentration of IL-10 (pg/mL). (c) Ratio of IL-12-p70/IL-10 calculated from absolute values measured for both cytokines in pg/mL and related to the particular experiment. (d) Concentration of TNF-*α* cytokine (pg/mL). (e) Concentration of IL-6 cytokine (pg/mL). (f) Concentration of IFN-*γ* (pg/mL). The *y*-axis represents the concentrations of cytokines in pg/mL. The results are expressed as mean ± SD of eight individual experiments. *P < 0.05; **P < 0.001 (significant difference to iDCs). Exact *P* values are given in Section 3.

It has been shown that DCs fundamentally affect naïve T lymphocyte development in different well-described subpopulations such as T helper cells including Th1, Th2, and Th17 types, T cytotoxic cells (Tc), and T regulatory cells (Tregs) [13]. T cell differentiation is conducted by DCs cytokine production. The cytokines IL-12 and IL-10 are critical in Th1 and Tregs cell differentiation. Considering that a predominance of either IL-12p70 or IL-10 is indicative of activatory or tolerogenic immune response mechanisms, we estimated the IL-12p70/IL-10 ratio by calculating the absolute values of concentration obtained from each experiment and for each particular DCs subtype. Thus, in aDCs, IL-12p70 expression constantly increased from 6 h aDCs (*P* < 0.05) to 24 h aDCs (*P* = 0.0005) when compared to iDCs (Figure 2(a)). The IL-12p70/IL-10 ratio in aDCs reflected the upregulation of IL-12p70 levels against IL-10 (Figure 2(c)). Nevertheless, in tDCs a reverse proportion between IL-12p70 and IL-10 was found. The IL-12/IL-10 ratio in tDCs clearly showed suppression of IL-12p70 production and upregulation of IL-10 cytokine during 24 h maturation (Figures 2(a)–2(c)). The tDCs and iDCs induced only slight TNF-*α*, IL-6, and IFN-*γ* cytokine expression in all time periods when compared to aDCs (Figures 2(d)–2(f)). Moreover, exposure to LPS and IFN-*γ* induced a strong production of proinflammatory cytokines TNF-*α*, IL-6, and IFN-*γ* at both 6 h and 24 h aDCs against tDCs and untreated iDCs control (Figure 2(d)). Taken together, these data indicate that LPS and IFN-*γ* trigger activation of human DCs while TGF-*β* and IL-10 trigger human DCs tolerogenic character during maturation* in vitro*.

### 3.3. miRNA Profiles in Human aDCs and tDCs in Comparison to Untreated DCs Control

To study miRNA level changes in generated DCs* in vitro*, we isolated miRNA enriched total RNA and performed quantitative PCR based on TaqMan low density array for miRNA expression analysis. The only observed changes in miRNA expression with fold change higher than twofold in all samples were analyzed.

**Figure 3:**
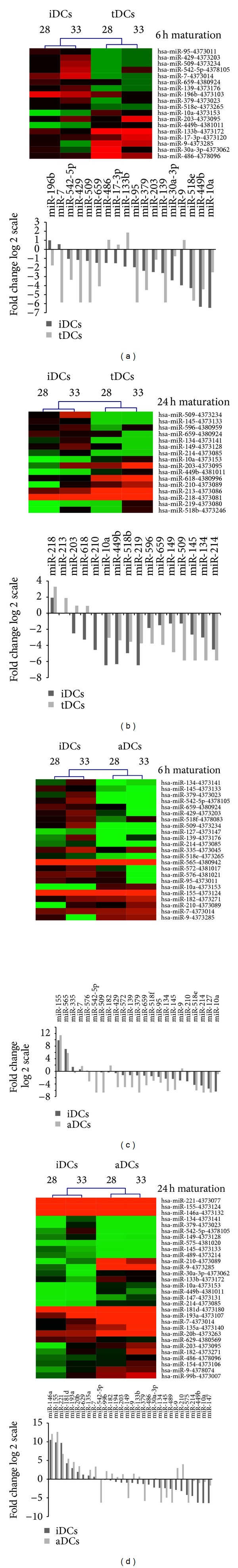
miRNAs profiles in activated and tolerogenic DCs in comparison to untreated DCs control. Immature iDCs were cultured with GM-CSF (800 UI/mL) and IL-4 (500 UI/mL) for 6 days. On day 6, the media were replaced for iDCs containing LPS/IFN-*γ* (aDCs) or containing IL-10/TGF-*β* (tDCs) for next 6 h or 24 h maturation. The q-PCR based TLDA miRNA expression analysis was provided in all DCs samples. The gene expression values for miRNAs were normalized using RNU6B as an endogenous control. The relative expression levels of target miRNAs were determined by the equation 2^−ΔCt^, in which ΔCt were calculated as follows: ΔCt = CtmiR-of-interest − Ct RNU6B. The observed changes in miRNA expression with fold change higher than twofold were analyzed. The results are visualized through heat maps and dendrograms. Green-red colour scale display in the heat maps increasing miRNA in measured samples. (a) miRNA expression in iDCs versus 6 h tDCs. (b) miRNA expression in iDCs versus 24 h tDCs. (c) miRNA expression in iDCs versus 6 h aDCs. (d) miRNA expression in iDCs versus 24 h aDCs.

**Figure 4:**
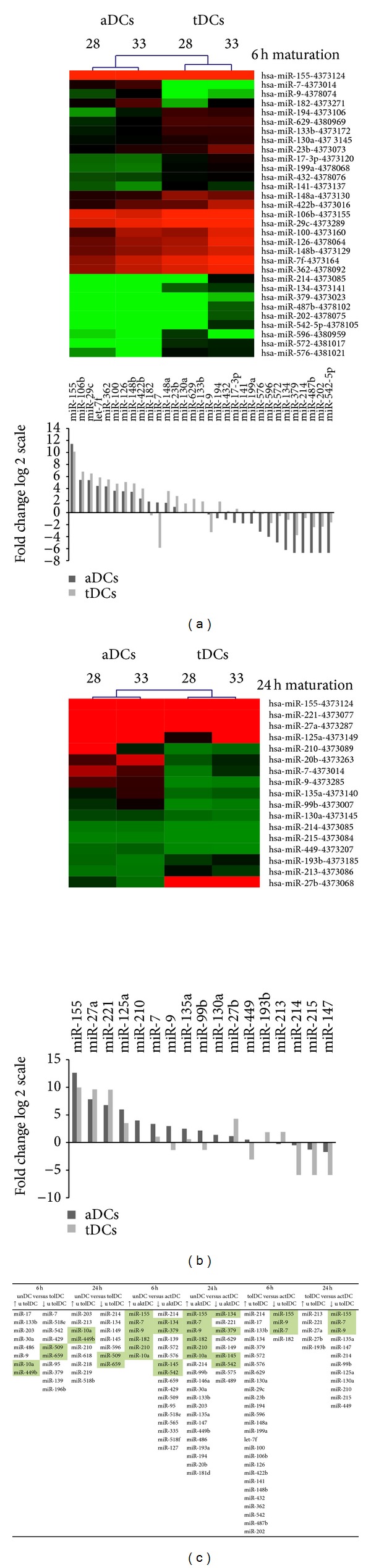
Comparison of miRNA profiles in activated versus tolerogenic DCs after 6 h and 24 h of maturation. Immature iDCs were cultured with GM-CSF (800 UI/mL) and IL-4 (500 UI/mL) for 6 days. On day 6, the media were replaced for iDCs containing LPS/IFN-*γ* (aDCs) or containing IL-10/TGF-*β* (tDCs) for next 6 h or 24 h maturation. The q-PCR based TLDA miRNA expression analysis was provided in all DCs samples. Gene expression values for miRNAs were normalized using RNU6B as an endogenous control. The relative expression levels of target miRNAs were determined by the equation 2^−ΔCt^, in which ΔCt were calculated as follows: ΔCt = CtmiR-of-interest − Ct RNU6B. The observed changes in miRNA expression with fold change higher than twofold were analyzed. The results are visualized through heat maps and dendrograms. Green-red colour scale display in the heat maps increasing miRNA in measured samples. (a) miRNA expression in 6 h aDCs versus 6 h tDCs. (b) miRNA expression in 24 h aDCs versus 24 h tDCs. (c) Table summarizing upregulated and downregulated miRNAs in aDCs and tDCs upon 6 and 24 h of maturation and iDCs.

Following 6 h of maturation with IL-10 and TGF-*β*, 7 miRNAs in tDCs were upregulated and 10 miRNAs downregulated compared with iDCs (Figures 3(a) and 4(c)). However, after 6 h of maturation by LPS and IFN-*γ*, aDCs exhibited 6 upregulated miRNAs and 17 downregulated miRNAs when compared to iDCs (Figures 3(c) and 4(c)).

Following 24 h of maturation, tDCs showed 9 upregulated miRNAs and 7 downregulated miRNAs compared with iDCs (Figures 3(b) and 4(c)). Comparing aDCs after 24 h with iDCs of maturation, aDCs upregulated 20 miRNAs and downregulated 9 miRNAs as shown in Figures 3(d) and 4(c). Interestingly, 5 miRNAs (miR-95, miR-429, miR-509, miR-542, and miR-659) were downregulated in tDCs and aDCs compared with iDCs.

We then compared the miRNA profiles in aDCs to tDCs after 6 and 24 h of maturation. Thus, after 6 h of maturation, we found expression changes in 31 different miRNAs. Among them, 27 miRNAs were upregulated in tDCs and only 4 miRNAs were downregulated in tDCs in comparison to aDCs (Figures 4(a) and 4(c)).

After 24 h of maturation, in tDCs only 5 miRNAs were upregulated whereas 12 were downregulated compared with aDCs (Figures 4(b) and 4(c)).

In total, these data indicate different miRNA profiles in aDCs and tDCs upon maturation* in vitro*. Four upregulated miRNAs are important for activation (miR-7, miR-9, miR-155, and miR-182) in aDCs compared to tDCs and iDCs after 6 h and 24 h of maturation. On the other hand, 4 different upregulated miRNAs (miR-17, miR-133b, miR-203, and miR-23b) are more important for tDCs induction than aDCs and iDCs after 6 h of maturation.

### 3.4. Correlation of miRNA Expression with Cytokine Production

According to the known role of selected miRNA, we analyzed the correlation between miRNA expression and cytokine production. We found a significant correlation between production of IL-12 and miR-221 expression (*p* = 0.0003; *r* = −0,9273) and a clear trend between IL-12 and miR-155 production. We did not find any correlation between IFN-*γ* production and expression of miR-29c.

## 4. Discussion

*In vitro* culture of DCs with demanded characteristics has made marked progress during the last decade. Not only different cultivation strategies and monitored DCs phenotype characteristics but also novel molecular evaluation methods have been developed. In this study, we aimed to connect phenotypes with miRNA profiling of* in vitro* prepared human blood monocyte-derived DCs from healthy subjects. To the knowledge of the authors, this is the first time human immature DCs subsequently affected by LPS and IFN-*γ* or by IL-10 and TGF-*β* for 6 or 24 hours have been evaluated using miRNA profiling.

At 6 h aDCs already strongly express costimulatory receptors CD80/CD86 together with high CD83 maturation marker necessary for T cell activation and survival [23]. The activatory phenotype of aDCs is enforced by the gradual growth of Th1 response promoter, IL-12, and proinflammatory cytokines, IFN-*γ*, IL-6, and TNF-*α*. To date, miRNAs have been described in regulating naïve and adaptive immune system response, immune cell development and differentiation, and prevention of autoimmune diseases [24–28]. By analyzing miRNA expression profiles in DCs using qRT-PCR, we identified 4 miRNAs (miR-7, miR-9, miR-155, and miR-182) uniquely overexpressed in aDCs treated for 6 h or 24 h with LPS and IFN-*γ* compared to untreated immature iDCs and to tDCs cultured with IL-10 and TGF-*β*. Recent investigations in different immune cell types have shown that TIRs and TNF-*α* receptor activation results in the rapid expression of miRNAs including miR-9, miR-99b, miR-146a, miR-146b, and miR-155 [29]. Specifically, the studies of LPS-induced miR-9, miR-146a, and miR-155 expression demonstrate a central role in the activity of the proinflammatory transcription nuclear factor- (NF-) *κ*B [29, 30]. Upregulation of miR-182, connected with the immune system, was referred to in sepsis patient leukocytes and activated helper T cells clonal expansion [31, 32].

Currently the most studied miRNA in DCs is miR-155. In our experiments, aDCs have upregulated miR-155, which agrees with the results obtained in some previous studies [33, 34]. Lu et al. found a correlation between the levels of miR-155 and miR-221 with IL-12 production, cell development, and proapoptotic effect in maturated DCs [33]. In our work we also found a significant correlation between the IL-12 production and the expression level of miR-221 and a trend with miR-155 level.

Furthermore, our data show that tDCs cultured for 6 h and 24 h with IL-10 and TGF-*β* induce decreased expression of surface markers CD80 and CD83 compared to aDCs and iDCs. The expression of HLA-DR molecule as a potential stimulator of T cell activation remained unaffected in our experiments in both tDCs and aDCs in agreement with other studies [35–37]. T cell stimulator CD86 molecule showed a trend in downregulating this marker in tDCs compared to aDCs. Both 6 h and 24 h tDCs slightly produce the proinflammatory cytokines TNF-*α*, IL-6, and IFN-*γ* together with restrained IL-12, which affects Th1 response in a negative manner and facilitates Th2 balance and T regulatory cells [38]. To the best of our knowledge, we here described, for the first time, the miRNA profiles in human tDCs stimulated for 6 h and 24 h with IL-10 and TGF-*β*. We found 3 miRNAs uniquely elevated in tDCs compared to iDCs and aDCs (miR-17, miR-133b, and miR-203) and miR-23b cluster (miR-23b and miR-27b). To date, miR-17 has been studied in the context of the autoimmune disease multiple sclerosis (MS) in which its expression is significantly reduced in peripheral leukocytes and overexpressed targeted miR-17 genes are involved in activating the immune system [39].

Our results also showed an elevated miR-133b level in tDCs. miR-133b function in immunocompetent cells is still very limited and the only reference deals with miR-133b expression in correlation with Th17 cell differentiation [40]. miR-379 was studied by Kallioniemi group in bone metastasis of breast cancer. Ectopic miR-379 expression in the cell line of breast carcinoma MDA-231 decreases the expression of genes including some involved in TGF-*β* signaling pathway [41]. In 6 h maturated tDCs we found decreased miR-23b, similar to Zhenga's results. His study on miR-23b tolerogenic character in DCs shows that its elevation is stimulated by ovalbumin. miR-23b expression inhibits the DCs maturation and reduced the DCs antigen uptake and also the expression of DCs surface markers [42]. Of note, our results show elevated miR-27b in 24 h tDCs expressed from the same cluster as miR-23b.

Importantly, our results showed 27 miRNAs (miR-17-3p, miR-23b, miR-29c, miR-100, miR-106b, miR-126, miR-130a, miR-133b, miR-134, miR-141, miR-148a, miR-148b, miR-194, miR-199a, miR-202, miR-214, miR-362, miR-379, miR-422b, miR-629, miR-432, miR-487b, miR-542-5p, miR-572, miR-576, miR-596, and let-7f) upregulated in tDCs cultured with IL-10 and TGF-*β* compared to aDCs stimulated with LPS and IFN-*γ* after 6 h of maturation. Interestingly, some miRNAs which were upregulated in tDCs (miR-23b, miR-27b, miR-10a, and miR-30a) are described as negative regulators of TGF-*β* signaling pathway [43], while miR-196, which is in our study downregulated in tDCs, is negatively regulated by TGF-*β* [44]. Ma et al. described the mechanism of IFN-*γ* regulation by miR-29. They found downregulation of miR-29 in activated natural killers, CD4+ T cells, and CD8+ T cells producing IFN-*γ* [45]. Moreover, miR-29 suppressed IFN-*γ* production by directly targeting IFN-*γ* mRNA. In our results, we did not find any correlation between miR-29c expression and the level of IFN-*γ*.

Furthermore, upregulation of the miR-148 family (miR-148a, miR-148b, and miR-152) was observed in DCs stimulated by LPS. The role of miR-148 family in activated DCs is to inhibit the MHC II expression, production of proinflammatory cytokines, and DCs-mediated CD4+ T cell expansion [46]. Let-7f was referred to as a potential regulator of IL-23 receptor expression in memory CD4+ T cells, which subsequently produce higher levels of IL-17 in comparison to naïve T cells [47].

On the other hand, we identified 4 miRNAs (miR-99b, miR-135a, miR-147, and miR-214) that were downregulated in 24 h tDCs when compared to 24 h aDCs. Similar miRNAs were upregulated in 24 h aDCs and iDCs. While upregulated miR-147 was identified in activated macrophages after multiple TLRs' stimulation, its expression is probably capable of downregulating excessive inflammatory responses [48].

Furthermore, we found 8 miRNAs (miR-95, miR-139, miR-379, miR-429, miR-509, miR-518e, miR-542-5p, and miR-659) downregulated in both 6 h aDCs and tDCs with respect to iDCs. We observed that, at 24 h of maturation, 5 miRNAs (miR-10a, miR-203, miR-210, miR-30a, and miR-449b) were upregulated in both tDCs and aDCs compared with iDCs while 3 miRNAs (miR-134, miR-145, and miR-149) were downregulated. Upregulated miR-10a was identified in T regulatory cells as a specific marker of these cells [49]. Another group found that TGF-*β* and retinoic acid induce the miR-10a expression, which targets Bcl-6 and constrains the plasticity of helper T cells [50]. Upregulated miR-203, miR-210, and miR-449b play a role in cell cycle control and inducting apoptosis. MiR-449b is induced by E2F1 and provides a safety mechanism to avoid excessive E2F1-induced proliferation by cell cycle arrest and apoptosis [51]. MiR-203 leads to G1 phase cell cycle arrest in laryngeal carcinoma cells by directly targeting survivin and miR-210 targets antiapoptotic Bcl-2 expression and mediates hypoxia-induced neuroblastoma cell apoptosis [52]. Conversely, cell function experiments have revealed that overexpression of miR-134 in A549 and Calu-3 cells can promote cell proliferation and inhibits cell apoptosis and migration abilities* in vitro* [53].

These miRNAs fine-tune immune response by cell cycle arrest and induce apoptosis of activated or tolerogenic cells to retain homeostasis of the immune system.

In conclusion, aDCs generated in the presence of LPS and IFN-*γ* for 6 h and 24 h displayed an immunoactivatory phenotype. miRNA profiling showed similar data to previous studies of proactivatory miRNAs [34]. Our results show that a minimum of 6 h treatment is sufficient to generate aDCs that are able to highly produce factors leading to Th1 response and that this capacity can be further extended for the next 24 h. We here describe the set of miRNAs induced in IL-10 and TGF-*β* cultured human blood monocytes-derived DCs. Finally, our results suggest that DCs miRNA profiling profitably supports DCs phenotype and functional studies. More intensive investigation of miRNA function in the future might bring greater insight into the regulation of immune systems and potential therapeutic possibilities of oncological or autoimmune disease.
